# Promoting scholarship in a community-based internal medicine residency

**DOI:** 10.1080/20009666.2018.1483692

**Published:** 2018-08-23

**Authors:** Joseph Fanciullo, Jennifer Hsu, Dennis C. Stevens

**Affiliations:** Internal Medicine Residency Program, University of South Dakota, Sanford School of Medicine, Sioux Falls, SD, USA

**Keywords:** Resident training, scholarship, research, evidence-based medicine, community-based internal medicine training program

## Abstract

The University of South Dakota Sanford School of Medicine Internal Medicine residency implemented a program to enhance scholarship among residents. This residency is part of a small Mid-Western community-based school.

**Background**: A Director of Research was hired and developed a structured approach consisting of: 1. Independent study regarding research methods and statistical testing and 2. Mentoring of residents and faculty in scholarly pursuits starting in the first months of residency.

**Methods**: Scholarship for two cohorts of residents for years July 2011–2014 and January 2014–2017 were followed. Products included papers accepted/published and papers accepted/presented at national or international meetings.

**Results**: 7 (14.8%) of 47 residents in the first cohort published 12 papers (0.25 papers/resident) with 18 faculty as co-authors (1.5/paper). 20 (43.4%) of 46 residents in the second cohort (structured program) published 39 papers (0.85 papers/resident) with 80 faculty as co-authors (2.1/paper). The difference in papers was significant by chi-square analysis.

**Conclusion**: A structured program requiring independent study in conjunction with individualized mentoring of scholarship starting early in the first postgraduate year was successful in significantly increasing the scholarly activity of our community-based internal medicine residents and faculty. With this program, the percentage of residents publishing exceeds national statistics recently reported.

## 1. Introduction

The Accreditation Council for Graduate Medical Education requires evidence of scholarship for faculty and residents [1]. In 2009, this was the main reason programs were cited for deficiencies. Numerous articles have been published regarding complex and costly methods to enhance scholarship [2–7]. The intent of the current paper is to discuss methods employed in a small, community-based internal medicine residency and to compare the results with recently published data.

The English Oxford Living Dictionary defines scholarship as ‘academic study or achievement’ or ‘learning at a high level’ [8]. Medical education and postgraduate education are characteristically scholarly activities. In medicine, scholarship is manifest in the: 1) Propagation of existing knowledge (teaching); 2) Discovery of new knowledge (research); 3) Review of new information by peers; 4) Dissemination of new knowledge at meetings and by publication; and 5) Application to practice with evidence-based practice. In community-based training, research opportunities are limited, and it may be difficult to stimulate faculty and learners to participate in scholarship beyond patient-based teaching.

Our Internal Medicine Residency was like other community-based programs in attempting to enhance scholarship without a major change in the complex training curriculum. Such enhancement is challenging because community-based faculty members are primarily clinicians who have little time to conduct research or publish. Productivity is based on the number of patients evaluated and revenue generated [9]. Additionally, clinicians may not perceive themselves as scholars or as mentors for learners and their experience in scholarship is frequently similar to that of the learner.

Prior to the changes outlined in this paper, an initial attempt was made to enhance scholarship by requiring second postgraduate year residents (PGY2) to prepare a poster presentation for the annual meeting of the state chapter of the American College of Physicians. Subsequent steps are described below.

## 2. Materials and methods

The residency appointed a director of research, who has a graduate degree and considerable experience in clinical research design and statistical analysis. The required qualifications and expectations for this position are listed in Table 1. No specific metrics were required for this position with the exception of annual reporting to the departmental chair. This paper is a detailed composite of these reports. Goals for this position were to prepare residents to:

1. Successfully respond to Board examination items regarding research design and statistical analysis;
2. Learn to interpret and critique the medical literature, to practice evidence-based medicine, to perform meaningful quality assurance, and;
3. Participate in scholarship with the mentorship of faculty resulting in publications and/or presentations at professional meetings.

**Table 1.** Research director qualifications and responsibilities.

**Qualifications:**
(1) Qualifications for appointment at the rank of Associate or Professor in School of Medicine.
(2) PhD or MD with extensive and ongoing research activity.
(3) Excellent communication skills.
(4) Commitment to education for medical students, residents and practicing physicians.
(5) Twenty percent time commitment.
**Responsibilities to the Department of Internal Medicine:**
(1) Review faculty appointments and make recommendations to the Appointment Committee.
(2) Mentor and assist faculty in research and scholarly activity.
(3) Participate in departmental and residency educational programs, committees and meetings.
(4) Provide a yearly written report to the chair summarizing resident scholarship.
**Responsibilities to the Internal Medicine Residency Program:**
(1) Accountable to the Residency Program Director for residents’ research experiences.
(2) Develop a curriculum which will provide residents the ability to successfully answer examination questions and to apply this knowledge to the review, interpretation and critique of the literature so as to achieve successful practice of evidence-base medicine.
(3) Match residents with faculty mentors for scholarly projects and research experiences.
(4) Counsel and mentor residents and faculty in the conduct of scholarly projects and research including issues in the use of human subjects, hypothesis development, research design, methods, data analysis, manuscript preparation and submission of scholarly articles for publication.
(5) Coordinate the research elective and participate in teaching and supervision of residents in the research setting
(6) Motivate clinical faculty and residents to actively engage in scholarly activities which are of interest to them.
(7) Mentor Residency Program faculty and residents to navigate through the IRB process, guide them through the research process and assist with writing to publication.

A self-study curriculum was designed on research. Selected resource materials for physicians were placed online through the medical library. During orientation, PGY1 residents take a baseline pretest on statistics and hypothesis testing and after studying the required materials, were required to score 75% on a post-test. Residents applied this information to review and critique published papers at the monthly journal club.

The following methods were used to facilitate scholarship:

1. Orientation of the PGY1 residents by the research director including a review of information regarding the self-study material, Collaborative Institutional Training Initiative (CITI, University of Miami) and EndNote reference manager software (Thomson Reuters).
2. Individual meetings (3–4) with the research director during the PG1 year to address:
  - Past experience in research;
  - Areas of interest;
  - Plans for subspecialty training;
  - Pairing with a faculty research/scholarship mentor;
  - Encouragement to embark on a literature review early in PGY1;
  - Repeat meetings to facilitate completion of projects, abstracts, presentations and papers.
3. Presentations by the research director at departmental meetings to encourage and mentor faculty scholarship.
4. Availability of the research director to assist faculty and residents in matters of research design, data management, data analysis, manuscript preparation and editing.

Resident scholarship was compared for the last two consecutive 3-year cohorts of residents. The first cohort began 1 July 2011 and completed 30 June 2014. The second cohort started 1 January 2014 through 31 December 2017. To account for delays in the review and publication process, products accepted or published for an additional 6 months, until 31 December 2014 for cohort 1 and 30 June 2017 for the second cohort, were included.

During the first cohort, residents did not have designated time for scholarship. In the second cohort, PGY1 residents had a 1-month research elective. After the first 2 years, the rotation schedule was changed so that residents have 2 days a week during 2-week outpatient rotations, occurring every 6 weeks [7].

Evidence of scholarship included papers accepted or published or papers accepted for or presented at national/international meetings. Posters for the state meeting were not included. Transitional residents were excluded but preliminary residents were included. The total numbers of publications/presentations, the numbers per resident and mean number of publications/presentations per resident were calculated. Chi-square testing [10] was performed comparing the proportions of residents with and without publications in the two periods. The number of authorships for residents planning and not planning fellowship training were compiled for the second cohort. The number of faculty co-authors was tallied.

## 3. Results

Prior to the implementation of the new research program, there were 47 residents and 7 (14.8% of residents) published 12 papers (0.25 papers/resident, Table 2). Following implementation of the new curriculum there were 46 residents. All successfully completed the online educational materials. Twenty (43.4%) residents published 37 papers and two presented abstracts at international meetings (rate 0.85 works/resident). The chi-square test was highly significant (*p* = 0.002) [10].

**Table 2.** Comparison of scholarship for two cohorts of residents.

Measure	Cohort 1	Cohort 2
Number of residents	47	46
Number publications*	12	39
Publications/resident	0.23	0.85
Residents published	7	20
% Residents published	14.8	43.4
Faculty co-authors	18	80
Faculty/paper	1.5	2.1
Residents planning Fellowship papers**	–	11/14 (78.6%)
Residents not planning Fellowship papers	–	7/32 (21.9%)

* *p* = 0.002, **p = 0.001

Of 14 residents who planned fellowship training, 11 (78.6%) published papers compared with 7 of 32 (21.9%) not planning further training (chi-square *p* < 0.001) [10]. Eighteen faculty members were co-authors of the 12 papers in the first cohort (1.5/paper) and there were 80 faculty co-authors of the 39 publications in the second cohort (2.1/paper).

## 4. Discussion

These results are attributable to the new curriculum and a transformation of the priority given to scholarship by the School of Medicine and the residency. Our scholarship program was adapted from similar programs previously described [2–7].

Traditional academic centres have reported a dramatic increase in publications and empiric research to nearly 100% with the implementation of a full research curriculum [6,7,11]. More frequent reports indicate that 25–35% of residents published or present papers during training [2,4]. Recent data regarding a large national survey of 252 internal medicine residency programs indicated that overall 36% of residents presented abstracts and 12% published manuscripts and 3% published book chapters. In regard to manuscripts published, 16% of residents in programs with a research track, 11% of those with protected time and 8% of residents in which scholarship was ‘encouraged’ published [12]. Our residents would have been classified as having protected time, but our numbers far exceed those reported with over 40% of residents having presented at international meetings (*n* = 2) or published manuscripts (*n* = 33).

We did not develop an extensive course but rather hold residents responsible for pertinent didactic material. We explore residents’ interests in scholarship, research and post-residency fellowship but are dependent upon the individual’s interests and motivation to produce results.

The research director plays an important role in the orientation, support of scholarship and for mentoring faculty, but is not involved in all products. Alternatively, an individual who is interested, but lacks extensive training in research, could fill this role but must be available to mentor faculty and trainees.

In spite of the noted success, challenges persist. There are a small number of faculty members involved in empiric research, although such opportunities are available for residents. The time of clinical faculty members to mentor is limited by their practice and the demands of teaching many learners [9].

Residents have very limited time due to heavy clinical training demands. In some instances, clinical training and preparation for examinations occupy too much time for scholarship. Frequently, residents elect not to participate in empiric research so as to concentrate on clinical training. Limited proficiency in English inhibits some international trainees. The greatest impediment to enhancing scholarship is the inherent lack of interest and/or motivation on the part of the resident [13].

Our data demonstrate that those who plan on subspecialty training produce more publications. This is not a new observation. Past investigations have clearly demonstrated that evidence of scholarship during residency is related to late scholarship produced following the completion of postgraduate medical education [11,14].

Some independent factors during the recent cohort may have enhanced scholarship. The state medical journal initiated a series of articles authored by residents and mentored by faculty. Residents are strongly encouraged to submit papers for this series, but few have. This has increased publication opportunities for all learners and co-authorships for faculty. It has also benefited faculty members pursuing academic promotion in the School of Medicine. As noted, twice the number of faculty were involved in publications as residents and faculty co-authorship has increased from 1.5 in the first to 2.1 per paper in the second cohort.

Case reports are often discouraged in academics [15]. These are the first level of inquiry and a valuable avenue for trainees and faculty explore scholarship [16–18]. Preparation provides rapid immersion into scholarship with external feedback. Such preliminary work may lead to subsequent case series, epidemiologic studies and perhaps randomized clinical trials. Learners get experience in working with co-authors and with journals in all stages of preparation.

Many medical professionals shy away from writing because it is not a skill developed through medical education [9]. Success with a focused task, and encouragement to keep writing, can be career changing for residents and those in teaching positions. Editorial assistance enhances the ability of residents to publish and has eliminated much of the frustration with writing and the preparation of manuscripts. This has been particularly important for those who do not speak English as a first language. It also provides reassurance for inexperienced faculty who are supervising resident publications.

Participation in scholarly projects enhances an individual’s self-satisfaction, self-esteem and satisfaction with residency training [19]. There are tangible personal benefits stemming from becoming the expert in a field, presenting the results of one’s own project, participation in national professional meetings and seeing one’s name in print. This training enhances the trainee’s capacity to practice evidence-based medicine [20] and to undertake meaningful quality assurance projects. Plus, there is benefit derived from having a professional diversion from the routine tasks of medical practice.

As with any research, there were limitations in this project. Authorships were tracked regardless of the resident’s rank among multiple authors. Tracking of resident and faculty publications was difficult. We did not address the issue of the quality or impact of the publications. In ours as in other community-based residencies, involvement in investigative work, which results in high-impact research is difficult to achieve and will generally need to be deferred until fellowship.

## 5. Conclusion

Attention to change is the first step to success in increasing the number of scholarly works produced by residents in internal medicine or any other field. We recommend that programs desiring to enhance this process consider the following:

1. Assign a faculty member to monitor and facilitate resident and faculty scholarship.
2. Set minimum requirements and provide resources for residents to develop the required understanding of the principles of research design, protection of human subjects, data management, statistical analysis and hypothesis testing.
3. Incorporate non-clinical time for resident scholarship.
4. Encourage residents to select topics of interest and complete a review of the literature early in PGY1.
5. Encourage and support writing skills for residents and faculty.
6. Encourage faculty participation as mentors of residents’ projects.
  - Mentor scholarship.
  - Encourage active participation in projects and writing.
  - Assist in matching with trainees with faculty.
7. Develop local opportunities for residents and faculty to present scholarly work.
8. Encourage local journals to dedicate space for authors in training and faculty members.
